# Investigation of Electrocardiographic Changes in Individuals with Three or More Cardiovascular Risk Factors on Santiago Island—The Cross-Sectional PrevCardio.CV Study

**DOI:** 10.3390/jpm14080876

**Published:** 2024-08-19

**Authors:** Patrícia Coelho, Kelly Mascarenhas, Júlio Rodrigues, Francisco Rodrigues

**Affiliations:** 1 Sport Physical Activity and Health Research & Innovation Center (Sprint), Polytechnic Institute of Castelo Branco, 6000-084 Castelo Branco, Portugal; patriciacoelho@ipcb.pt; 2 Polytechnic Institute of Castelo Branco, 6000-084 Castelo Branco, Portugal; a2022123467@estesc.ipc.pt; 3 Instituto Nacional de Saúde Pública de Cabo Verde, Cidade da Praia 7600, Cape Verde; julio.m.rodrigues@insp.gov.cv

**Keywords:** adults, prevalence, cardiovascular risk factors, electrocardiogram, pathology, Cabo Verde

## Abstract

Cerebrocardiovascular diseases represent one of the greatest public health concerns globally. In Cabo Verde, non-communicable diseases, such as cerebrocardiovascular diseases, have become leading causes of morbidity and mortality. This study aimed to correlate risk factors with cardiac electrical changes in adult individuals residing on Santiago Island—Cabo Verde. A cross-sectional population-based study using simple random sampling was conducted in 2021 with individuals aged 18 and over, of both sexes, having authorization 35/2021 from the Cabo Verde Ethics Commission. The sample size was calculated based on Santiago Island’s projected population for 2021, considering an estimated prevalence of 50%, a 95% confidence interval, and a standard error of 4%, resulting in a sample of 599 individuals. The data were collected through a questionnaire on risk factors and cerebrocardiovascular diseases, blood pressure measurement, capillary blood glucose evaluation, and a 12-lead electrocardiogram. The study sample was predominantly female (54.8%), with the largest age group being 18–27 years (21%). Among the sample, 9.3% had no risk factors, 27.5% had one risk factor, 36.2% had two risk factors, and 26.9% had three or more risk factors. Of those who underwent electrocardiography, 60.24% showed electrocardiographic changes, with the most prevalent being ventricular repolarization changes, nonspecific repolarization changes, and early repolarization. A relationship was observed between cerebrocardiovascular disease risk factors and the electrocardiographic changes found in the study participants.

## 1. Introduction

Cerebrocardiovascular diseases (CCVD) constitute one of the major concerns in health and economy [1]. These pathologies cause comorbidities that significantly affect the quality of life of affected individuals and their families [2]. It is estimated that more than four out of five deaths from CCVD are due to cardiac events and strokes, with a significant portion of these deaths occurring in young adults [3,4]. According to the recent data, CCVD remain the leading cause of death globally, with approximately 18 million deaths each year, accounting for nearly 32% of all deaths worldwide [5,6]. This burden is especially pronounced in low- and middle-income countries, where healthcare resources are often limited [7,8].

The resting electrocardiogram (ECG) records the cardiac electrical potential captured through surface electrodes. It is one of the essential diagnostic tools used in various clinical settings, particularly in emergency contexts, primarily for acute coronary syndrome, pre-surgical evaluation, in patients with risk factors, diagnosed heart conditions, and in athletes [9]. The ECG is a non-invasive, cost-effective, and easily accessible test, making it indispensable in the early detection and management of cardiovascular diseases. Epidemiological studies have shown that resting ECGs can predict mortality and cardiovascular events in asymptomatic adults. The prevalence of ECG abnormalities rises significantly with age, from approximately 10% in individuals aged 35 to 25% by the age of 60, highlighting the importance of regular cardiovascular screening as the population ages [10,11,12].

Ventricular repolarization abnormality is an ECG anomaly highly associated with cardiovascular mortality. The analysis of the ST-T segment is critical, as alterations in this segment often indicate injury or obstruction in the coronary arteries. The research has shown that common ECG changes in stroke patients include QT interval prolongation, ST segment depression or elevation, U wave appearance, T wave inversion or peaked T waves, increased P wave amplitude, elevated QRS voltage, and the appearance of Q waves. Hypertension (HTN) is strongly linked to left ventricular hypertrophy (LVH), a major indicator of cardiovascular events such as sudden death and myocardial infarction (MI) [9,11,13]. Obesity, excessive salt intake, genetic predisposition, and diabetes contribute to these abnormalities by promoting blood pressure increases and cellular changes that lead to LVH. Diabetes, in particular, has been shown to cause abnormalities in ventricular depolarization or repolarization, with QTc interval prolongation being a precursor to malignant ventricular arrhythmias [14].

Although various diagnostic methods are available for heart diseases, the ECG remains a primary tool due to its simplicity, practicality, and cost-effectiveness. The resting ECG, in conjunction with symptomatology, is crucial for diagnosing heart diseases, monitoring cardiac electrical changes, and assessing prognosis, making it a first-line test in many clinical scenarios. Furthermore, the ability to perform ECGs in a variety of settings, including primary care and remote locations, underscores its versatility and utility in managing cardiovascular health [9,10].

Santiago Island, the most densely populated area in Cape Verde, comprises nine municipalities with diverse characteristics and a wide range of CCVD risk factors, reflecting the country’s broader reality. The population of Santiago Island faces unique challenges due to socioeconomic and lifestyle factors that influence the prevalence and management of CCVD.

The study highlights the clinical importance of diagnosing and monitoring CCVDs, using resting ECG as a key tool. The novelty lies in the application of this knowledge to a specific population, allowing a detailed analysis of how multiple risk factors impact cardiovascular health in a unique regional context.

## 2. Materials and Methods

### 2.1. Type of Study

An observational, cross-sectional, prospective, analytical, population-based study was conducted.

### 2.2. Participants

- Location and sample:
  ○ The sample was proportionally distributed among the various municipalities on Santiago Island, with the data collection procedure being performed randomly and at home.
  ○ The study sample was calculated based on the demographic projections for Cabo Verde from 2010 to 2030, which showed that there were 317,238 individuals on Santiago Island in 2021.
  ○ For the sample calculation, an estimated prevalence of 50%, a 95% confidence interval, and a 4% standard error were considered. The sample size is 599 individuals distributed across the nine municipalities on the island under study.
- Inclusion and exclusion criteria:
  ○ Inclusion criteria: individuals over 18 years old, of both sexes, residing on Santiago Island, and who agreed to participate in the study by signing the informed consent.
  ○ Exclusion criteria: Cabo Verdean nationals who had been abroad for more than a year and had returned to Santiago Island less than 30 days before the start of the study. Also excluded were individuals with any disability that might have prevented them from participating in the study.

### 2.3. Variables

- Data collect:
  ○ The individuals were invited to participate after reading the informed consent form, with all procedures explained. The households were selected by random sampling, and contact with the individuals began with an index house, from which nearby residences were surveyed, alternating three or more houses in any direction whenever possible. In residences where more than one individual met the inclusion criteria, data collection procedures were applied to all.
  ○ A cardiovascular risk factors assessment questionnaire was applied.
  ○ The collected data included: age, sex, race, weight, height, presence of diabetes, dyslipidemia, family history, physical activity, alcoholism, smoking habits, history of cardiovascular events, weight and blood pressure monitoring habits.
  ○ Physical exercise: quantified by frequency (number of days per week) and duration (minutes/hours) of activity.
  ○ Smoking habits: assessed by the number of cigarettes smoked daily and the number of years of use.
  ○ Alcohol consumption: quantified by the number of glasses consumed per day, during and/or outside of meals.
  ○ History of cardiovascular conditions: participants were asked if they had experienced myocardial infarction (MI), stroke, or transient ischemic attack (TIA).
  ○ Family history: participants were inquired about the existence of any relatives with known heart disease.
  ○ Monitoring habits: participants were asked about their habits of monitoring blood pressure and weight and how frequently they did so.

### 2.4. Measurements

- Weight and height:
  ○ Weight was measured using a SECA^®^ digital scale (Hamburg, Germany) with participants instructed to stand on the scale barefoot, with empty pockets, upright posture, and arms along the body.
  ○ Height was measured using a SECA^®^ portable stadiometer (Hamburg, Germany) placed on a flat surface. Participants were instructed to remove any objects from their head that might interfere with the measurement, to stand with their back to the stadiometer, legs and feet parallel and arms along their sides.
  ○ BMI: categorized according to the WHO guidelines, using the formula BMI = weight/(height)^2^ [15].
- Blood pressure:
  ○ Blood pressure assessment was carried out following the protocol of the Beira Baixa Blood Pressure Program (PPABB) using an automatic OMRON M3^®^ device (Kyoto, Japan). Three readings were taken, with additional readings if the first two measurements varied by more than 10 mmHg. Blood pressure values were classified according to the 2023 European Society of Cardiology Guidelines [16].
- Diabetes:
  ○ The presence of diabetes was verified through capillary blood glucose measurement conducted at the time of the questionnaire, using a manual glucose meter with test strips from the FreeStyle^®^ brand (Abbott Park, IL, USA). The values were analyzed according to data from the Portuguese Diabetes Protection Association [17].
- Lipid sheet:
  ○ Assessed through a questionnaire regarding knowledge of cholesterol levels, triglycerides, and history of medication use for cholesterol reduction.
- Electrocardiographic evaluation:
  ○ The electrocardiographic evaluation was performed on participants who presented three or more associated risk factors, using the Schiller^®^ electrocardiograph model AT101 (Baar, Switzerland). A 12-lead ECG was performed, with the individuals remaining at rest for at least 3 min, lying on a bed, in the supine position, with their muscles relaxed, arms stretched along the body and legs extended. Monitoring was performed in the conventional manner, using 4 peripheral electrodes placed on the participants’ limbs, namely, R—right arm, L—left arm, F—left leg, N—right leg (neutral). The precordial electrodes were placed in the precordial region, with V1 in the fourth intercostal space on the right margin of the sternum, V2 in the fourth intercostal space on the left border of the sternum, V3 in the middle between electrodes V2 and V4, V4 in the fifth left intercostal space on the midclavicular line, V5 at the same level as electrode V4 on the anterior axillary line, and V6 at the same level as electrodes V4 and V5 on the midaxillary line. After all electrodes were placed and checked, the electrocardiographic recording was started with a graph paper speed of 25 mm/s and amplitude of 1 mV per 10 mm. The analyses of the electrocardiographic recordings were performed based on the recommendations or the standardization and interpretation of the electrocardiogram guidelines (75–78). The electrocardiographic parameters were analyzed according to normality requirements, namely, heart rate (50–100 bpm); heart rhythm; electrical axis (from −30° to 90° normal axis, −30° to −90° axis deviated to the left, 90° to 180° axis deviated to the right, −90° and −180° extreme deviation of the electrical axis); P wave (positive or biphasic polarity), amplitude (≤2.5 mm), and duration (0.08 and 0.12 s); QT interval (350 ms to 450 ms); PQ segment duration (0.12 to 0.20 s); QRS complex duration (≤0.120 s), and amplitude (≤25 mm). The existence of alterations in the ST segment was analyzed, being normal when isoelectric. It was also verified whether the T wave was positive in all leads except aVR and the existence of a pathological Q wave.

### 2.5. Statistical Analysis

- Software and tests used:
  ○ The data were analyzed using the IBM SPSS Statistics^®^ software (Statistical Package for the Social Sciences), version 20.
  ○ To assess the normal distribution of the sample, the Kolmogorov–Smirnov normality test was applied.
  ○ Associations between variables were examined using the chi-square test, with a significance level of *p* ≤ 0.05 and a 95% confidence interval.

The consort diagram makes it clear that the entire process was successful, with no loss of participants at any stage of the study. It transparently demonstrates that the 599 individuals initially selected were followed until the end of the study and had their data analyzed, which strengthens the reliability of the study results.
Assessment for eligibilityEvaluatedExcludes5990Selection of participantsEligible ParticipantsParticipants who agreed and were included599599Data collectParticipants who completed data collectionParticipants who did not complete data collection5990Data analysisIncluded in the final analysisExcluded in the final analysis5990Consort diagram.

## 3. Results

The study sample consisted of 599 individuals of both sexes, all of Black race, with females predominating at 54.8%, compared to 45.2% for males. The sample was collected from the nine municipalities on Santiago Island. The minimum age of respondents was 18 years and the maximum were 90 years, with an average age of 44 years ± 17.06 years. The age group with the highest number of individuals was 18 to 27 years, representing 21% of the total.

A BMI value ≥ 25 kg/m^2^ was considered a risk factor for CCVD. It was found that 9.3% of the respondents were underweight, 48.1% had normal weight, 27.2% were overweight, and 15.4% were obese.

**Figure 1:**
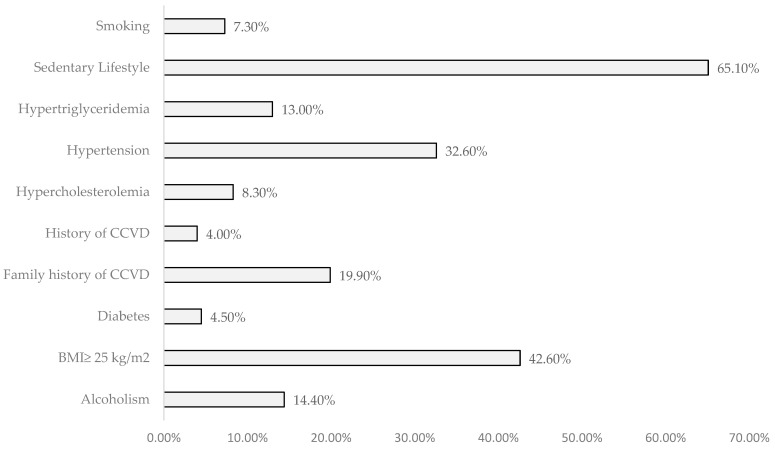
Prevalence of Studied Risk Factors. Legend: BMI—body mass index; CCVD—cardiovascular diseases.

Regarding physical activity, it was observed that 34.9% of the sample engaged in exercise with an average frequency of 4 times per week and an average duration of 59 min and 46 s. Sedentary behavior was more common among females (60.5%), showing a very significant statistical relationship (*p* < 0.001, Table 1). Although there was no statistically significant relationship with BMI (*p* = 0.145), the analysis of Figure 1 indicates that there are more sedentary individuals with a BMI ≥ 25 kg/m^2^ compared to those with normal weight. Additionally, among the active individuals, the majority had normal weight (54.1%).

Regarding smoking as a risk factor, it was found that 7.3% were current smokers, 3.7% were ex-smokers, and 4.7% used narcotics, with the age group 28 to 47 years (53.48%) having the highest tobacco consumption. The average number of years smoking among the smokers was 16.8 years.

In the study of alcohol consumption, it was found that 14.4% reported daily drinking habits, and 37.1% had infrequent habits (only on weekends/celebrations). A more detailed study of this habit showed that daily alcohol consumption averaged approximately 3.7 glasses, with 92.7% consuming alcohol during and/or outside of meals.

When related to educational level, it was observed that both smoking and alcohol consumption were more prevalent among individuals with basic education, aged 28 to 47 years, and males, showing a statistically significant relationship (*p* < 0.001).

Analyzing the lipid and diabetic profiles assessed through the questionnaire, it was found that 7.5% of individuals reported a diagnosis of diabetes, 8.3% had a diagnosis of hypercholesterolemia, 1.3% had hypertriglyceridemia, and 12% reported a history of dyslipidemia.

Of the 7.5% of diabetic individuals, 11.9% were insulin-dependent, 26.2% followed a diet, and 61.9% were treated with pills. Of the 72 individuals who had hypercholesterolemia, 61.4% were on pharmacological treatment, 11.4% managed it with diet alone, and 27.1% did not monitor it at all.

Diabetes, hypercholesterolemia, hypertriglyceridemia, and a history of hypercholesterolemia showed higher prevalence in females and the age group 58 to 67 years, with a statistically significant relationship, except for hypertriglyceridemia.

For capillary glucose assessment, values were classified as hypoglycemia, normal glucose, pre-diabetes, and diabetes. Glucose values ranged from 389 mg/dL to 32 mg/dL, with an average of 104.95 mg/dL. Of the evaluations conducted, 89.8% were postprandial and 10.2% fasting. It was found that 7.7% had pre-diabetes values and 4.5% had values corresponding to diabetes. There was a higher predominance of females and the age group 58 to 67 years in both pre-diabetes and diabetes.

To determine systolic blood pressure (SBP) and diastolic blood pressure (DBP), the average of the three blood pressure measurements was taken. SBP ranged from 211.47 mmHg to 76 mmHg, with an average of 128.47 mmHg, and DBP ranged from 150 mmHg to 51.67 mmHg, with an average of 82.75 mmHg. It was found that the prevalence of hypertension in the adult population of Santiago Island is 32.6%. According to the distribution of the sample by age group, hypertension was more prevalent in the 68 to 77 years group, followed by the 58 to 67 years group, showing a statistically significant relationship (*p* < 0.001).

Regarding the history of cardiovascular diseases among individuals, it was noted that 3.5% of respondents reported having had a transient ischemic attack/stroke, with 66.7% of these being females in the 58 to 67 years age group. In the study of myocardial infarction (MI), 3 cases (0.5%) were identified, all of which were female.

The hereditary risk factor was also studied, revealing that 19.9% of individuals reported having close family members with cardiovascular conditions, although 66% could not specify the exact conditions of their relatives.

From the study of risk factors analyzed, it is observed, according to Figure 1, that sedentary behavior, BMI ≥ 25 kg/m^2^, and hypertension had the highest prevalence rates.

In the follow-up of this study, the distribution of the number of risk factors for CCVD among individuals was analyzed. It was observed that only a minority had no risk factors for CCVD, while the majority had 2 or 3 risk factors, as can be seen in Table 1.

Electrocardiograms were performed on all individuals with three or more associated risk factors, representing 26.9% of the sample, totaling 161 electrocardiograms, with the highest prevalence in the age group of 48–67 years (47.2%), showing a statistically significant relationship (*p* < 0.001).

In the analysis of the electrocardiogram parameters, the following were considered: heart rhythm, heart rate (HR), P wave (amplitude and duration), QT interval, cardiac axis, PQ interval, QRS complex (duration and amplitude), T wave, and the presence of Q and U waves.

The minimum heart rate found was 40 bpm and the maximum was 117 bpm, with an average of 76 bpm, and 95% of the individuals had a heart rate within the normal range (Table 2).

Of the electrocardiograms performed, 96.9% showed sinus rhythm, while 3.1% exhibited atrial fibrillation, atrial tachycardia, and sinus arrhythmia. The electrical axis was within the normal range in 93.8% of the ECGs, as can be observed in Table 3.

Regarding the P wave, 95% of the electrocardiograms had amplitude values within the normal range (≤2.5 mm), while 5% had increased amplitude (≥2.5 mm), with 87.5% of these cases being female. The duration of the P wave was increased (>0.12 s) in two individuals, one female and one male.

All ECGs had the QT interval within the normal range (0.35–0.45 s), and 93.8% had the PQ segment within the expected range (0.12–0.20 s). Among those with an altered PQ segment, 1.2% had a decreased segment and 4.9% had an increased segment, with the majority being female.

No cases of ST segment elevation or depression were observed; 98.8% of the ECGs had the QRS complex within the normal range (≤0.12 s). Only one individual, female, had an increased QRS amplitude (>25 mm).

In the analysis of the T wave, 16.1% of the ECGs did not show a positive T wave in all leads except aVR, with 69% of these cases being female. Pathological Q waves were found in 3.1% of individuals, with the majority being female (80%).

Electrocardiographic changes were observed in 60.24% of the ECGs and were categorized into ventricular repolarization abnormalities (negative T waves, flattened T waves), nonspecific repolarization changes, early repolarization, first-degree atrioventricular block, T-peak waves, intraventricular disturbances (complete right bundle branch block, incomplete right bundle branch block, left anterior hemiblock), atrial hypertrophy (right and left), sinus tachycardia, ventricular extrasystoles (interpolated, isolated), left ventricular hypertrophy, atrial tachycardias (atrial fibrillation), supraventricular extrasystoles, and other changes (bradycardia, transition abnormalities, respiratory arrhythmia, Q wave). Table 4 describes the electrocardiographic changes found, and it can be noted that the most frequently recorded changes were in ventricular repolarization, followed by nonspecific ventricular repolarization changes and early repolarization, with a higher prevalence in females.

**Figure 2:**
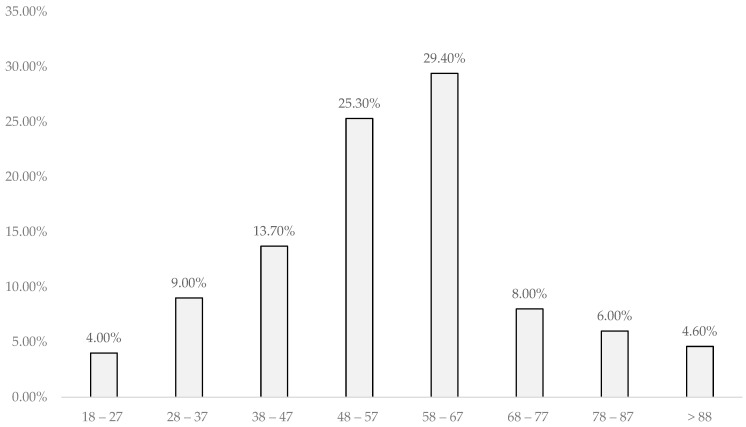
Distribution of Electrocardiographic Changes by Age Group. Legend: %—percentage.

According to the number of individuals in each age group, the highest prevalence of electrocardiographic changes was found in the age group above 88 years (50%), followed by 78–87 years (35%), showing a statistically significant relationship (*p* < 0.001), as analyzed in Figure 2.

Electrocardiographic changes were also related to all the risk factors studied, with a statistically significant relationship observed for all of them except for smoking, as shown in Table 5.

## 4. Discussion

The electrocardiogram is a useful tool for diagnosing and monitoring cardiac conditions, being easy and quick to perform, low-cost, and considered a predictive means of cardiovascular events [18]. Various factors can interfere with the conduction of the electrical impulse and consequently reflect in possible alterations in the ECG. A total of 161 ECGs were performed, 99 on females and 62 on males. Of the ECGs performed, 60.2% showed electrocardiographic changes, with the majority of the alterations (56.7%) found in females and a higher prevalence in the age group over 88 years. The research conducted by Brito, Franciele et al. [19] in Brazil revealed 40.23% of ECGs with electrocardiographic changes. In another study conducted by Araya, Ma et al. [20] in Asians, ECG changes were found in 79.1% of individuals. The differences observed across studies may be associated with genetics, race, and study methodology.

The study conducted by Sebold, Fábio et al. [21] in Brazil found a prevalence of electrocardiographic changes at 20.2%, with these being more associated with sex, alcoholism, hypertension, and diabetes. This prevalence is lower than that found on the island of Santiago, a difference that may be due to the study population, geographic area, and age range in the Brazilian study, which was between 19 and 59 years.

The most predominant alterations in the population of Santiago Island were ventricular repolarization changes, followed by nonspecific repolarization changes and early repolarization. Vinculação, Rosimara et al. [22] studied 3341 Brazilian individuals and found 76.6% of ECGs with alterations, with the most predominant being nonspecific repolarization changes. The research conducted by Samu Porto Alegre, Brazil, led by Maciel, Ana et al. [23], which included 1338 ECGs, identified predominant changes such as sinus tachycardia, ST-segment elevation, atrial fibrillation or flutter, right bundle branch block, and anteroseptal block. In Brazilian studies, regarding the most prevalent alterations, only nonspecific repolarization changes were common with the findings from Santiago Island; however, these differences can be explained by the aforementioned factors

Left ventricular hypertrophy is characterized by an increase in the thickness of the left ventricular muscle walls, often as a consequence of hypertension. Black individuals have a greater predisposition to developing hypertension, with factors such as low birth weight and less nocturnal blood pressure reduction possibly explaining this predisposition [24]. Hypertension promotes the development of LVH, which occurs as a compensatory mechanism to the hemodynamic overload caused by increased blood pressure in the arteries in order to offset the high pressure on the ventricular wall, thereby preserving the mechanical function of the left ventricle and cardiac output. Myocardial fibrosis caused by LVH facilitates the development of heart failure (HF), malignant ventricular arrhythmias, and sudden death [25,26]. Given the high incidence of HTN in the black population, a high prevalence of LVH would be expected, which was not observed in this study. Only two individuals with ECG criteria for LVH were found on Santiago Island: one male and one female. In Angola, Gonçalves, Mauer et al. [27] found a prevalence of LVH of 37.2%, with a predominance in males. The higher prevalence of LVH found in Angola is likely due to the larger sample size, a possibly higher prevalence of HTN, and other factors mentioned earlier, as well as the fact that on Santiago Island, ECGs were performed only on individuals with three or more associated risk factors.

Atrial fibrillation (AF) is defined as a supraventricular arrhythmia caused by electrophysiological changes that trigger ectopic foci in the atria and pulmonary veins, leading to an increased heart rate and irregular rhythm. This condition is more common in the elderly, males, and those with a history of cardiovascular disease. Studies have shown that AF predominantly arises due to cardiovascular risk factors and that the arrhythmia itself promotes the occurrence of cerebrocardiovascular events, such as stroke/transient ischemic attack (TIA) and myocardial infarction (MI) [28,29]. In the results found, only one individual undergoing an ECG had AF, and this individual was female. Other studies conducted in the black population have also reported a low incidence of AF. In South Africa, Gonçalves, Mauer et al. [27] found an AF prevalence of 2%. However, according to Zoni Berisso et al., the results from hospital studies conducted in South Africa, Senegal, Burkina Faso, and Cameroon showed slightly higher prevalence rates than in the studied island, at 4.6%, 5.4%, 5.9%, and 7.1%, respectively [30]. Similarly, another study conducted in Ethiopia, which studied individuals aged ≥40 years, found a prevalence of 4.3% [31].

The electrocardiographic changes found in the adult population of Santiago Island showed a statistically significant relationship with all the analyzed risk factors, namely, sex, obesity, physical inactivity, alcohol consumption, hypercholesterolemia, hypertriglyceridemia, hypertension, and diabetes, except for smoking.

The work conducted by Crisostomo, Luciola et al. [32] in Brazil, involving 220 patients (110 diabetic), found that electrocardiographic changes were significantly greater in diabetics, with the main changes being ventricular repolarization alterations (31.8%), arrhythmias (21.8%), left ventricular overload (14.5%), left anterior fascicular block and right bundle branch block (both 7.3%), and left atrial overload (6.4%).

In the study by Bessaria, Valéria et al. [33] conducted at a hospital in Brazil, individuals with hypertension, those on antihypertensive medications, and diabetics showed electrocardiographic changes in 14.5%, 16.3%, and 21.5%, respectively. In this Brazilian study, males showed a higher predominance of changes but without statistical significance. This discrepancy between the Brazilian study and the Santiago Island study is likely related to geographic area, race, sample size, and method of data collection, with the Brazilian study being conducted in an emergency hospital setting.

Risk factors are associated with both cerebrocardiovascular morbidity and mortality. It is crucial to raise awareness among the population about controlling weight, blood pressure, blood glucose, and performing routine ECGs to detect changes that could help prevent cardiovascular events or their worsening.

The findings underscore the need to strengthen health promotion strategies so that individuals, families, and communities can better engage in maintaining their health, adopting healthy habits and lifestyles, and implementing preventive measures, particularly for cerebrocardiovascular conditions.

The resting electrocardiogram (ECG) is a valuable tool in clinical practice, particularly for screening individuals with suspected cardiovascular (CV) disease. It is widely used because it is non-invasive, low-cost, and easy to perform, providing important information about the heart’s electrical activity.

One of the main uses of the resting ECG is its ability to identify individuals with a lower likelihood of coronary artery disease (CAD). In individuals presenting with symptoms suggestive of CV disease but with a resting ECG showing no significant abnormalities, the likelihood of significant CAD can be reduced. This is because many forms of CAD, especially less severe ones, may not produce detectable changes on a resting ECG. Thus, a normal resting ECG can help rule out significant CAD, allowing doctors to better structure patient management and treatment.

Furthermore, a resting ECG can provide prognostic information about the progression of patients over time. Patients with a normal ECG generally have a better prognosis in terms of future cardiac events compared to those with electrocardiographic abnormalities. In this way, the resting ECG can assist in risk stratification, helping to identify patients who need more detailed evaluation and those who can be monitored with less intensity.

Several studies have attempted to make associations and predict future situations based on exams, aiming to increase the ability to anticipate cardiovascular events [34].

Based on the discussion presented, the main differences between the results obtained in the population of Cape Verde (Santiago Island) and populations from other regions are the following: in Cape Verde, 60.2% of electrocardiograms (ECGs) showed changes, with a higher prevalence in women and those over 88 years of age; while in Brazil, the study by Brito et al. revealed 40.23% of ECGs with changes; while Sebold et al. found a prevalence of 20.2%, more associated with sex, alcoholism, hypertension, and diabetes; and in the Asian Population, Araya et al. found 79.1% of ECGs with alterations, a higher prevalence than in Cape Verde, suggesting genetic, racial, and methodological differences [19,20].

Regarding the type of electrocardiographic changes, in Cape Verde, the most predominant changes were changes in ventricular repolarization, followed by nonspecific changes and early repolarization; and in Brazil, Vinculação et al. found 76.6% of ECGs with changes, the most common being nonspecific repolarization changes; and for Maciel et al., the predominant changes included sinus tachycardia, ST segment elevation, atrial fibrillation/flutter, and bundle branch blocks, which differs from the most common changes found in Santiago [22,23].

In left ventricular hypertrophy (LVH), in Cape Verde, only two individuals presented LVH criteria on the ECG, and in Angola, Gonçalves et al. found a prevalence of LVH of 37.2%, predominantly in men, a significant difference that can be explained by the sample size and the prevalence of hypertension [27].

In atrial fibrillation, in Cape Verde, only one individual was identified with AF, being female, and in other African populations, AF prevalences varied between 2% (Gonçalves et al., South Africa) and up to 7.1% (Cameroon), suggesting a generally low prevalence but still higher than that observed in Santiago [27,30].

The discrepancy in the prevalence and types of changes can be attributed to geographic, racial differences, sample size, and data collection methods, with the Santiago Island study involving a population sample, while some Brazilian studies were carried out in emergency hospital contexts.

These differences highlight the importance of considering factors such as genetics, environment, and research methods when interpreting the results of electrocardiographic studies in different populations.

## 5. Conclusions

The most prevalent electrocardiographic changes in individuals with three or more risk factors were ventricular repolarization abnormalities, nonspecific repolarization changes, and early repolarization, with a higher predominance in females and primarily in individuals over 78 years old.

It is important to perform routine electrocardiograms for the early detection of changes that can help prevent major cardiovascular events and, thus, reduce the comorbidity and mortality associated with cerebrocardiovascular risk factors.

## 6. Limitations

Based on the general analysis of this study, the main limitations relate to several factors. Variables such as family history of heart disease, alcohol consumption habits, and smoking were obtained through self-reported questionnaires, which are subject to memory bias or social desirability bias. This can affect the accuracy of the collected data. Although the ECG is an important diagnostic tool, it cannot detect all cardiac conditions or provide a complete picture. Certain abnormalities may go undetected or be misinterpreted, especially without the use of complementary diagnostic techniques such as echocardiograms or Holter monitoring.

Comparisons with studies from other populations (Brazil, Angola, Asia, etc.) are limited due to differences in data collection methods, as well as geographical, demographic, and clinical contexts. Variations in the prevalence of electrocardiographic abnormalities may be influenced by these factors and may not necessarily reflect intrinsic differences between populations.

The study’s cross-sectional design limits the ability to establish causal relationships between risk factors and electrocardiographic abnormalities. The study captures only a specific moment in time, making it impossible to track the progression of cardiac conditions over time.

These limitations should be considered when interpreting the study’s results and in planning future research to explore more deeply the characteristics and risk factors of the Cape Verdean population. Nevertheless, the study provides extremely valuable information, particularly for the fields of epidemiology and public health, in a geographical area where investment in these components is still not very strong.

## Figures and Tables

**Table 1:** Distribution of Number of Risk Factors per Individuals.

Distribution RF	n	Total (%)	Sex M (%)	Sex F (%)	Predominance AG
None RF	56	9.3	58.9	41.1	18–27
One RF	165	27.5	47.3	52.7	18–27
Two RF	217	36.2	45.2	54.8	38–47
Three or more RF	161	26.9	38.6	61.4	58–67

Legend: RF—risk factors; n—number of individuals; %—percentage; M—male; F—female; AG—age group.

**Table 2:** Evaluation of Heart Rate of Individuals Who Underwent Electrocardiogram.

Heart Rate	n	Total (%)	Sex F (%)	Sex M (%)
Bradycardia (<50 bpm)	3	1.9	33.3	66.7
Normal (50–100 bpm)	153	95	62.1	37.9
Tachycardia (>100 bpm)	5	3.1	60	40

Legend: n—number of individuals; bpm—beats per minute; %—percentage; F—Female; M—Male.

**Table 3:** Evaluation of cardiac electrical axis.

Electrical Axis	n	Total (%)	Sex F (%)	Sex M (%)
Normal (−30° to 90°)	151	93.8	80	20
Left Axis Deviation (−30° to −90°)	8	5	75	25
Right Axis Deviation (90° to 180°)	2	12	0	100

Legend: n—number of individuals; %—percentage; F—Female; M—Male; BPM—Beats per minute.

**Table 4:** Distribution of Electrocardiographic Changes Found.

Electrocardiographic Changes	n	Total (%)	Predominant Sex
Ventricular Repolarization Changes	26	26.8	Female
Nonspecific Repolarization Changes	13	13.4	Female
Early Repolarization	12	12.4	Male
First atrioventricular	8	8.2	Female
Big T-wave	7	7.2	Female
Intraventricular disturbance	7	7.2	Female
Atrial hypertrophy	6	6.2	Female
Sinus tachycardia	4	4.1	Same
Ventricular extrasystole	3	3.1	Male
Left ventricular hypertrophy	2	2.1	Same
Atrial tachycardia	2	2.1	Male
Supraventricular extrasystole	1	1.0	Female
Other Changes	6	6.2	Male

Legend: n—number of individuals; %—percentage.

**Table 5:** Relationship Between Electrocardiographic Changes and Risk Factors.

Risk Factors	*p*
Sex	0.028
BMI ≥ 25 kg/m^2^	<0.001
Sedentary Lifestyle	<0.001
Alcoholism	0.027
Smoking	0.352
Hypercholesterolemia	<0.001
Hypertriglyceridemia	<0.023
Family history of CCVD	<0.001
Hypertension	<0.001
Diabetes	<0.001
History of CCVD	0.007

Legend: BMI—body mass index; CCVD—cerebrocardiovascular diseases.

## Data Availability

The original contributions presented in the study are included in the article, further inquiries can be directed to the corresponding author.
